# Choices: The Science of Bela Julesz

**DOI:** 10.1371/journal.pbio.0020172

**Published:** 2004-06-15

**Authors:** Ralph M Siegel

## Abstract

Highlights of Bela Julesz's scientific career in visual neuroscience

Throughout his career, Bela Julesz created new scientific disciplines by remarkable combinations of seemingly disparate approaches. The selection of his major discipline, which would eventually be called visual neuroscience, may have been serendipity or choice.

When the unexpected Soviet invasion of Hungary in 1956 spurred his emigration to the United States, Bela Julesz, with his Hungarian doctorate in engineering, joined the numerous mathematical luminaries working at AT&T Bell Laboratories, such as John Tukey, Harry Nyquist, Claude Shannon, and John Kelly. One of the projects underway at the time was the creation of long random-number binary sequences that did not repeat. Bela told the story that he was assigned the problem of testing these number generators; he decided to use the best pattern recognizer that he knew of—the human visual system. The random bits of zeros and ones drawn from the random number sequences were plotted as sequential rows in an image. Any repeats, any correlations across space, would be instantly seen by the human visual system as patterns in the random dots. What caused Bela to choose this unusual approach to looking for patterns, combining computers and vision? His doctoral thesis research in network theory and television signals clearly influenced him, but it was quintessential Bela to give himself a hand up into a new field by building on his base of knowledge, moving in a new and unexpected direction using mathematical and psychological insight. He termed this talent “scientific bilingualism” (Julesz 1994).[Fig pbio-0020172-g001]

**Figure pbio-0020172-g001:**
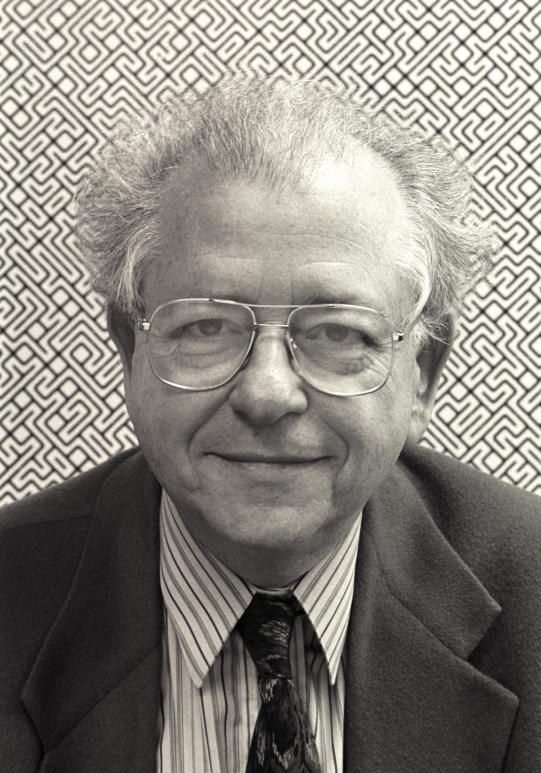
Bela Julesz, in front of a picture from his and A. Michael Noll's computer art exhibition, “Computer-Generated Pictures,” held at the Howard Wise Gallery, New York City, in 1965. Photograph courtesy of Rutgers University

This success in exploiting the visual system, and the intellectual freedom intrinsic to the design of Bell Labs, provided Bela with the opportunity to use these new random dot patterns to explore the visual system. Most of us know well that we can use the small differences in the images in each eye to see depth. Sir Charles Wheatstone showed in 1838 that if two different perspective images were observed through a stereoscope so that each eye observed only one view, a startlingly realistic three-dimensional image occurred. Oliver Wendell Holmes, stereoscope enthusiast, wrote of the experience that “the shutting out of surrounding objects, and the concentration of the whole attention, which is a consequence of this, produce a dreamlike exaltation…in which we seem to leave the body behind us and sail away into one strange scene after another, like disembodied spirits” (Holmes 1861).

The basis of this three-dimensional perception was hotly debated between Wheatstone and fellow physicist Sir David Brewster. (Though it may seem odd for physicists to concern themselves with the physiology of optics, this was felt to be a natural extension of the study of the physics of optics.) Brewster opined that perspective was the source of the apprehension of an object's shape. Wheatstone insisted that the images in the each eye had identifiable landmarks that were combined to assign depth to the landmarks. Bela read much of the literature of that time, and he must have seen two greats as wrestling without either finding the overwhelming hold to pin down the other. More than one hundred twenty years after Brewster and Wheatstone, Bela realized that his random dot patterns could be used to probe this question. What Bela did was create a pair of identical random dot patterns. When viewed binocularly through a stereoscope (i.e., fused), they would be seen as a single surface. Then Bela took a central region from the right random dot pattern and displaced it minutely to the right. Now when the two patterns were fused, the central square was not seen double, but after a moment or two, eerily moved into depth, behind the surrounding region. In 1960, Bela's experiment with what eventually became known as Julesz random dot stereograms unambiguously demonstrated that stereoscopic depth could be computed in the absence of any identifiable objects, in the absence of any perspective, in the absence of any cues available to either eye alone. It was a perfect combination of psychological and mathematical insight and technology that solved this puzzle. (It is an interesting aside that Bela sent his first report to the *Journal of the Optical Society of America*, where it was rejected; the *Bell Labs Technical Journal* holds the now classic paper [Julesz 1960]. The *Journal of the Optical Society of America* published Bela's second paper [Julesz 1963].) The stereoscope had existed 125 years.

Bela proposed in his book *Foundations of Cyclopean Perception* (1971) that early in the vision process the two images from the two eyes were combined to form a single view, imbued with inherent depth information. The perceptual “cyclops within us” was proposed to analyze the visual world first, before the motion, color, and contrast systems began their perceptual operations. Bela's book is full of powerful visual experiments that make this point irrefragably; from his psychophysical analysis, binocular vision forces unexpected constraints on the rest of vision, *Q.E.D. Foundations of Cyclopean Perception* is still considered one of the classics of modern psychophysics and continues to have profound relevance to both those entering the field and established investigators—over thirty years after its publication. At the time of his death, Bela had begun working on a second edition.

His success in determining the sequence of visual processing using random dot stereograms led Bela to propose that the anatomical hierarchy of the visual system could be understood in part through visual psychophysics—he termed this approach “psychoanatomy.” His ingenious use of the stereogram established a new approach in the field of vision research and presaged the now common use of carefully controlled computational techniques in brain science. By this time Bela's reputation was established, and in 1983, he received a prestigious MacArthur Fellowship—the “genius award.” He used the funds for travel, including an annual peregrination to the California Institute of Technology, where I first met Bela in 1985.

His seminars and lecture courses were enthusiastically received and endorsed by countless students, post-doctoral trainees, and faculty, as evidenced both by his formidable reputation and through the numerous citations of his work. His approach to presenting his research was modest and gently self-deprecating. He always encouraged young scientists; his joy and passion in their science were transmitted both through his warm persona and his suggestions of directions for future study. His insights guided my development of random dot kinematograms (i.e., movies) to examine how motion could be used to construct three-dimensional form (Siegel and Andersen 1988). He collaborated with Derek Fender, David Van Essen, and John Allman at the California Institute of Technology on the combination of the computer, the psychophysical approach, and the physiological experiment.

Bela was a fount of ideas, each building on the prior's advance. His later passions were explorations of texture and attention, notably with Jonathan Victor and Dov Sagi. Bela's appealing hypothesis that textons (putative elements of textures) are represented at a cellular level is now questionable (Julesz et al. 1978). Bela was groping for an overarching computational theory for the representation of random geometry, but none was to be had. Nonetheless, the texton elements served useful duty in the demonstration that there were two stages to early vision—an effortless phase preceding attention and a guided identification phase (Sagi and Julesz 1985). Many contemporary laboratories examining vision, studying either perception or the activity of neurons, now incorporate designed, complicated, yet highly controlled stimuli that have evolved (knowingly or not) from Bela's original forays in the 1960s and 1970s. His continuing impact was recognized by his election to the National Academy of Science in 1987.

In 1989, Bela retired from Bell Labs (by then he was a department head) and joined the Department of Psychology at Rutgers University to establish the Laboratory of Vision Research. Bela continued investigating mechanisms of form, texture, and stereopsis; his presence led to numerous studies into the implications of his original findings as well as new investigations into computational vision. His collaborations greatly aided the establishment of neuroscience at Rutgers. Bela wrote *Dialogues on Perception* (1995), a wide-ranging intellectual effort, in which he uses classic dialectics to question both his own successes and those of his chosen field. In the book one reads of two competing intellects, a Bela who believes in his contributions to science and another Bela who is constantly belittling and judging his contributions.

Throughout his career Bela Julesz was able to add language after language to his research imperative, becoming a true scientific polyglot. Although his arrival in the United States was propelled by political events beyond his control, his intellectual directions followed a chosen path “less traveled by, and that has made all the difference.” In 1956, an engineer set out from Hungary. By 2003, his unique combination of mathematical precision combined with deep biological insight had carried him to elegant solutions for seemingly intractable problems in visual neuroscience. Bela was always in dialogue, often with others, and often with himself. In the process, he would gently drive each of us, and himself, forward to our final destination of understanding the brain. Bela Julesz died on December 31, 2003, forty-seven years to the day after starting at Bell Laboratories.
